# Role of Minor Groove Width and Hydration Pattern on Amsacrine Interaction with DNA

**DOI:** 10.1371/journal.pone.0069933

**Published:** 2013-07-29

**Authors:** Deepak K. Jangir, Suman Kundu, Ranjana Mehrotra

**Affiliations:** 1 Quantum Optics and Photon Physics, National Physical Laboratory, Council of Scientific and Industrial Research, New Delhi, India; 2 Department of Biochemistry, University of Delhi South Campus, New Delhi, India; The Scripps Research Institute, United States of America

## Abstract

Amsacrine is an anilinoacridine derivative anticancer drug, used to treat a wide variety of malignancies. In cells, amsacrine poisons topoisomerase 2 by stabilizing DNA-drug-enzyme ternary complex. Presence of amsacrine increases the steady-state concentration of these ternary complexes which in turn hampers DNA replication and results in subsequent cell death. Due to reversible binding and rapid slip-out of amsacrine from DNA duplex, structural data is not available on amsacrine-DNA complexes. In the present work, we designed five oligonucleotide duplexes, differing in their minor groove widths and hydration pattern, and examined their binding with amsacrine using attenuated total reflection Fourier transform infrared (ATR-FTIR) spectroscopy. Complexes of amsacrine with calf thymus DNA were also evaluated for a comparison. Our results demonstrate for the first time that amsacrine is not a simple intercalator; rather mixed type of DNA binding (intercalation and minor groove) takes place between amsacrine and DNA. Further, this binding is highly sensitive towards the geometries and hydration patterns of different minor grooves present in the DNA. This study shows that ligand binding to DNA could be very sensitive to DNA base composition and DNA groove structures. Results demonstrated here could have implication for understanding cytotoxic mechanism of aminoacridine based anticancer drugs and provide directions to modify these drugs for better efficacy and few side-effects.

## Introduction

Topoisomerase enzymes are ubiquitous in nature because they play important task of regulation of superhelicity of DNA. Many biologically significant phenomena like DNA replication, transcription, segregation and recombination required topoisomerase enzymes [1]-[3]. In vertebrates, topoisomerase type 1 and type 2 are found. In its course of action, topoisomerase 2 creates double strand breaks in opposite strands of DNA, passes an intact segment of DNA from this transient opening and finally reseals these openings in DNA. This process, according to need of cell, either decatenates or changes topological number of DNA by ±2 [4]–[7]. Topoisomerase 2 wrapped DNA fragments are short lived and generally termed as cleavable complex [8]. Stabilization of these cleavable complexes by different topoisomerase 2 poisons including amsacrine though, results in their accumulation in the cell. These stabilized ternary complexes act as replication hurdles and stall the movement of replication machinery [9]. This inhibition of replication process activates various DNA repair mechanisms but high steady-state concentrations of these ternary complexes result in permanent double strand breaks and other mutational errors which in turn finally lead to cell death [1], [3], [8]. Hence, the critical phenomenon behind the poisoning of topoisomerase 2 by different antitumor agents is stabilization of the cleavable complexes [10].

**Figure 1:**
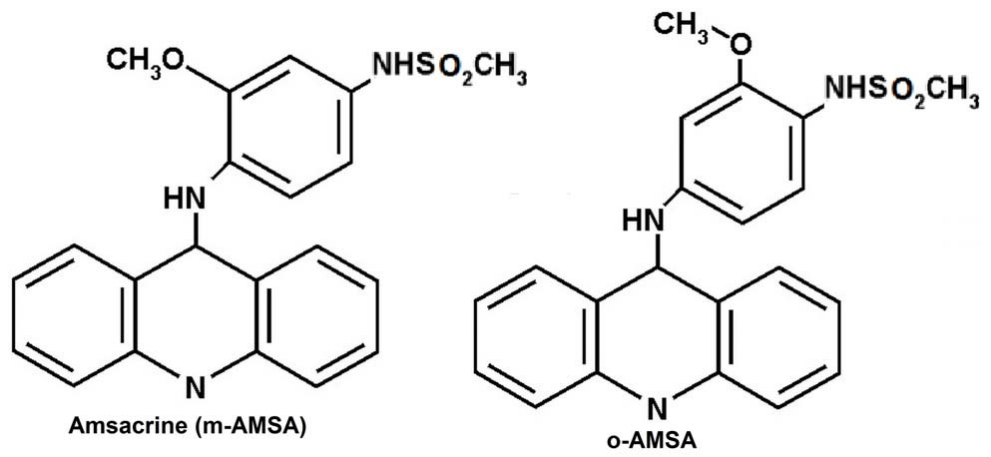
Structure of (a) amsacrine (m-Amsa) and (b) o-Amsa.

Amsacrine is the first anilinoacridine based chemotherapeutic drug (Figure 1), which is used to treat a broad spectrum of malignancies including acute leukemias and lymphomas [11]–[13]. Topoisomerase 2 is a natural target of amsacrine and related compounds in cells and thus forms topoisomerase 2 mediated double-strand break of DNA [14], [15]. In its course of action, amsacrine forms ternary complex with DNA and topoisomerase 2. Recent studies show that amsacrine is sandwiched between DNA binding sites and topoisomerase 2 enzyme [16], [17]. Thus, in ternary complexes, topoisomerase 2 and amsacrine share common binding sites on DNA and amsacrine directs the enzyme’s binding on the DNA duplex [16], [17]. Therefore, interaction of amsacrine with DNA duplex is crucial determinant of topoisomerase 2 mediated DNA cleavage pattern.

Despite a direct association between DNA binding properties of aminoacridine derivatives and topoisomerase 2 toxicity, DNA adducts of amsacrine and related compounds are not characterize yet. The major factor behind the difficulty to obtain information on amsacrine-DNA complexes is reversible binding of amsacrine to DNA with a transition time of ∼1–6 ms [18]. Due to this short transition time of binding, there is no X-ray crystallographic, NMR spectroscopy or electrophoresis record available on amsacrine-DNA complexes. Complete understanding on DNA binding mechanism of amsacrine could be helpful to resolve many unrequited questions associated with the amsacrine mediated topoisomerase 2 toxicity and may also provide an input for new drug designing. For instance, it is not known why the compounds which share common aminoacridine chromophore with amsacrine and differ only by side chains, exhibit different DNA binding properties and consequently produce very diverse DNA cleavage patterns induced by topoisomerase 2 [19], [20]. Likewise, o-AMSA, which is an isomer of m-AMSA (Figure 1) (thus shares same reactive groups) is biologically inactive [20], [21]. Further, it is unclear why m-AMSA appears to specifically stimulate DNA cleavage by Topoisomerase 2 at sites in DNA adjacent to thymine or adenine residues and whether there is a preference for binding of the methoxyaniline moiety of the amsacrine in the major or minor groove [18], [22], [23]. Sequence preference of amsacrine with DNA binding is also not well defined.

In the present work, we utilized attenuated total reflection–Fourier transform infrared (ATR-FTIR) spectroscopy technique to study DNA binding behavior of amsacrine. FTIR spectroscopy has an edge over other mentioned techniques that it does not require stable complex formation between ligand and biomolecule therefore information can be drawn on transiently bound complexes. FTIR spectroscopy is increasingly used for biomolecule-ligand interaction studies and for identification of conformations of biomolecules [24]–[29]. High level sensitivity and the potential to distinguish different conformational sub-states of biomolecules simultaneously in solutions are the merits of ATR-FTIR spectroscopy which enabled us to illustrate the detailed information on DNA binding properties of amsacrine.

## Experimental Section

### Sample Preparation

Calf thymus DNA (ctDNA) type I (Molar excitation coefficient ε = 6600 cm^−1^) and amsacrine were procured from Sigma-Aldrich chemicals, USA. Five different oligonucleotide duplexes (RP-HPLC purified), varying in their nucleobases and their sequential arrangements [(AAAAAAAAAAAA)2 ε = 174828, (ATATATATATAT)2 ε = 190086, (TTAATTAATTAA)2 ε = 182385, (AGAGCAACAGAG)2 ε = 189879 and (AGAGACCAGAGA)2 ε = 193683] were purchased from Eurogenetec, Belgium and Sigma-Aldrich chemicals, USA. ctDNA and oligonucleotides were dissolved in 100 mM Tris-HCl buffer (pH 7.4). Concentration of solutions of ctDNA and duplexes were checked spectrophotometrically using their excitation coefficients as provided by the manufacturers. The final concentration of ctDNA and duplexes in their stock solutions was adjusted to 20 mM. Molar ratios (drug/nucleotide) of 1/10, 1/20 and 1/50 were prepared by incubation of varying amount of amsacrine with constant ctDNA and duplex concentrations.

### FTIR Spectra

FTIR Spectra were recorded on Bruker Tensor series 32 spectrophotometer. Spectral measurements were performed after 2 hr incubation of amsacrine with ctDNA and duplexes. Freshly prepared samples were used for all the measurements. The solution spectra were recorded using Miracle® (Pike) ZnSe HATR crystal. Two hundred fifty six scans were co-accumulated in the spectral range of 2400-600 cm^−1^ with a resolution of 2 cm^−1^. Background atmospheric spectrum was collected before every measurement. A spectrum of buffer solution was recorded and subtracted from the spectra of free ctDNA, duplexes and their complexes with amsacrine to perform water subtraction according to established procedure [30]. For each measurement, three independent samples were used and their spectra were subsequently averaged to ensure the reproducibility of results. Sample chamber was continuously purged with dry nitrogen to remove water vapor. All the spectral measurements were carried out at room temperature. No data treatment was performed except multiple baseline correction and normalization for 1085 cm^−1^ band. Second derivatives were calculated using Savitzky-Golay functionality.

## Results

Five dodecamer oligonucleotide duplexes having varied base sequence were designed: (AAAAAAAAAAAA)2, (ATATATATATAT)2, (TTAATTAATTAA)2, (AGAGCAACAGAG)2, and (AGAGACCAGAGA)2. These duplexes and ctDNA were incubated with different molar ratios of amsacrine to obtain their complexes. Wavenumber assignments to IR bands of ctDNA and oligonucleotides are according to literature [28], [31]-[34]. Second derivatives of all the spectra are generated for better differentiation between overlapping bands and to compare intensity variations. Spectra of free duplexes show canonical B-DNA conformation. Though, upon careful examination of the region 950-700 cm^−1^, it is apparent that duplexes differ slightly in some spectral features. For instance, spectra of duplex (AGAGCAACAGAG)2 and (AGAGACCAGAGA)2 exhibit subtle N-type sugar conformation features (∼860 cm^−1^ and ∼808 cm^−1^). Presence of these features is consistent with the given fact that increasing content of G/C base pairs favor N type sugar conformation in DNA [33], [34]. Spectral presence of these minute features signifies the single nucleotide level sensitivity of ATR-FTIR approach that is also supported by some earlier observations [28], [34].

### Amsacrine-DNA Complexes

**Figure 2:**
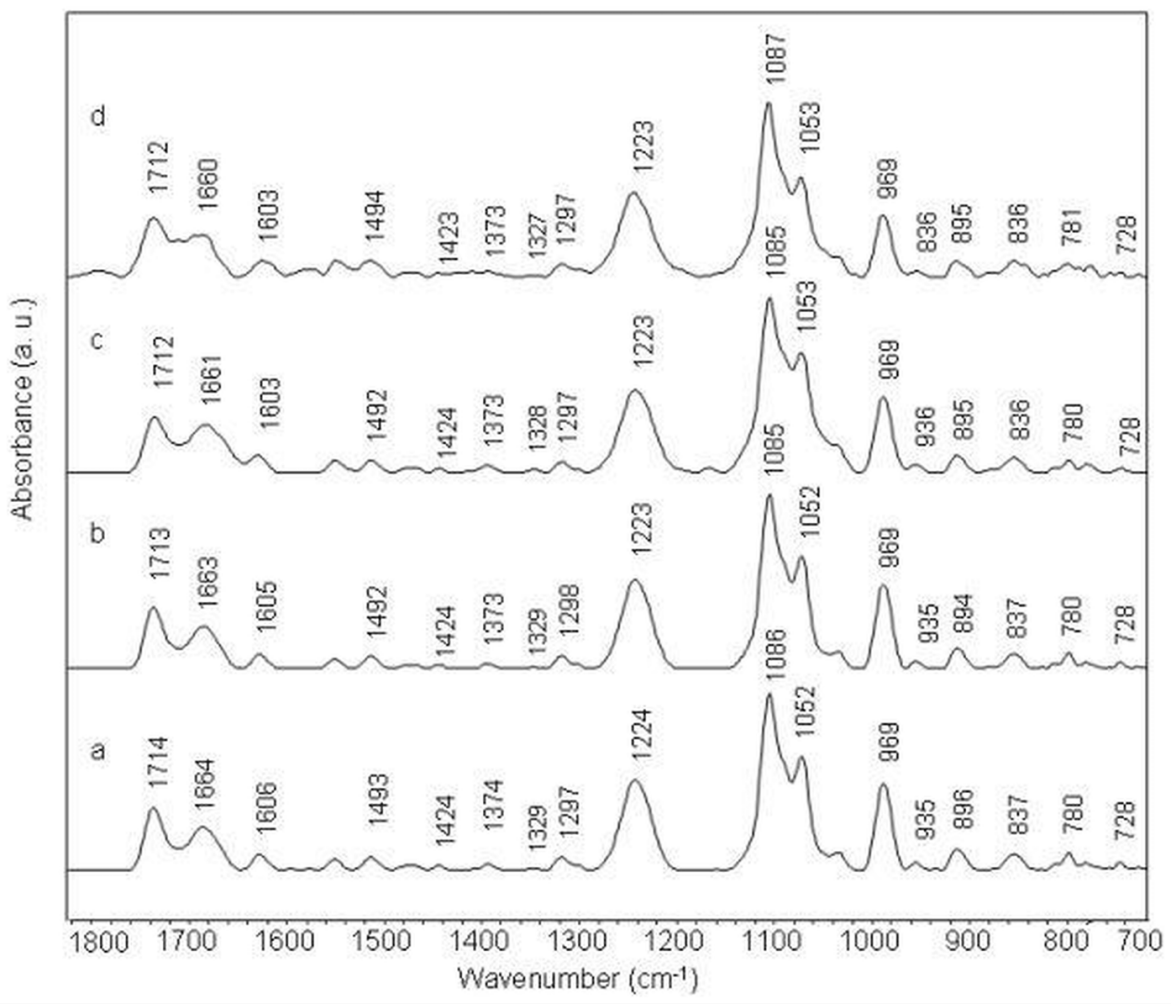
Stacked view of FTIR spectra of ctDNA (a) and its complexes with amsacrine at different molar ratios (r) of 1/50 (b), 1/20 (c) and 1/10(d).

**Figure 3:**
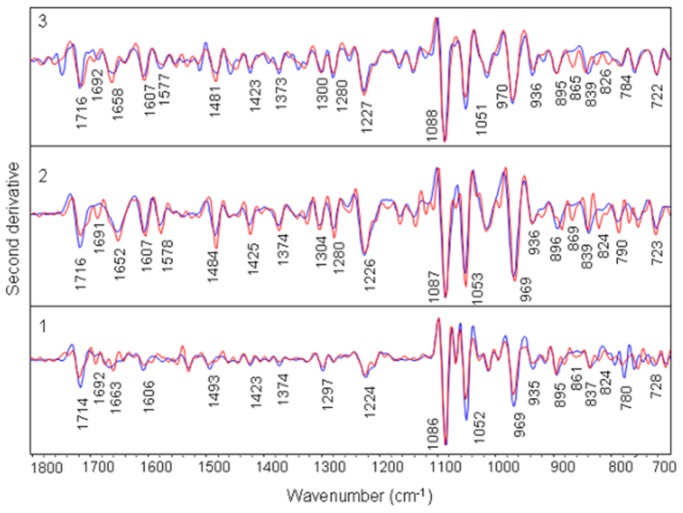
Overlaid view of second derivative spectra of ctDNA and duplexes with their complexes of amsacrine at molar ratio of 1/10 (drug/nucleotide). Panel 1: ctDNA (blue) and complex (red), Panel 2: (AAAAAAAAAAAA)2 (blue) and complex (red) (b), Panel 3: (ATATATATATAT)2 (blue) and complex (red), Panel 4: (TTAATTAATTAA)2 (blue) and complex (red), Panel 5: (AGAGCAACAGAG)2 (blue) and complex (red), Panel 6: (AGAGACCAGAGA)2 (blue) and complex (red).

Infrared band at 1714 cm^−1^ in the spectrum of free calf thymus DNA (ctDNA) is assigned to in-plane stretching vibrations of guanine and adenine aromatic rings. This band shifts to 1712 cm^−1^ in amsacrine-ctDNA complex at 1/10 molar ratio (Figure 2). Thymine C2 = O2 stretching vibration (with some contributions of C4 = O4 stretching) band assigned at 1664 cm^−1^ and adenine ring stretching band at 1606 cm^−1^, shift to 1660 cm^−1^ and 1603 cm^−1^ respectively in drug-DNA complexes. These bands also exhibit intensity variations with amsacrine addition to ctDNA (Figure 3 panel 1). Guanine band (1614 cm^−1^) and adenine band (1606 cm^−1^) show slight reduction in intensity while thymine band (1664 cm^−1^) exhibits some increase in absorbance. Cytosine band ct 1493 cm^−1^ did not exhibit any appreciable shift though; slight intensity change is observed for this band ((Figure 3 panel 1)). Moderate shift and intensity changes for IR bands of adenine thymine base pairs (at 1660 cm^−1^ and 1606 cm^−1^) are assigned to intercalation between these base pairs.

There are well-established infrared marker bands for intercalation and groove binding modes of interaction [26], [30], [35], [36]. Moderate shifts and intensity variations in guanine and cytosine bands (at 1715 cm^−1^ and 1493 cm^−1^ respectively) are assigned to intercalation of ligand between these base pairs of DNA while major groove binding is characterize by change in intensity and position shift in 1715 cm^−1^ band [26]. Cytosine band remains intact in this interaction. Similarly, moderate shifting and intensity changes for infrared marker bands assigned to adenine thymine base pairs signify intercalation between these base pairs. Thymine oxygen group (C2 = O2) is present in minor groove of DNA. Stretching vibrations of this group is assigned around 1690 cm^−1^ in the infrared region which is a specific marker for minor groove binding interaction [35], [36]. Any ligand interaction at minor groove results in intensity changes for this peak. Intercalation does not affect the peak.

In the amsacrine-DNA complexes, a low intensity band around 1692 cm^−1^ is evident at 1/10 amsacrine DNA molar ratio (Figure 2). This band is overlapped by surrounding high intensity bands around 1712 and 1660 cm^−1^; however it is clear in the second derivative spectra (Figure 3 panel 1). As aforementioned, this band is specifically assigned to vibrations of thymine C2 = O2 moiety, which lies in DNA minor groove [35], [36]. Changes in this band are associated with ligand interaction (through hydrogen bond probably) with thymine C2 = O2 group present in the minor groove of DNA [35], [36].

**Figure 4:**
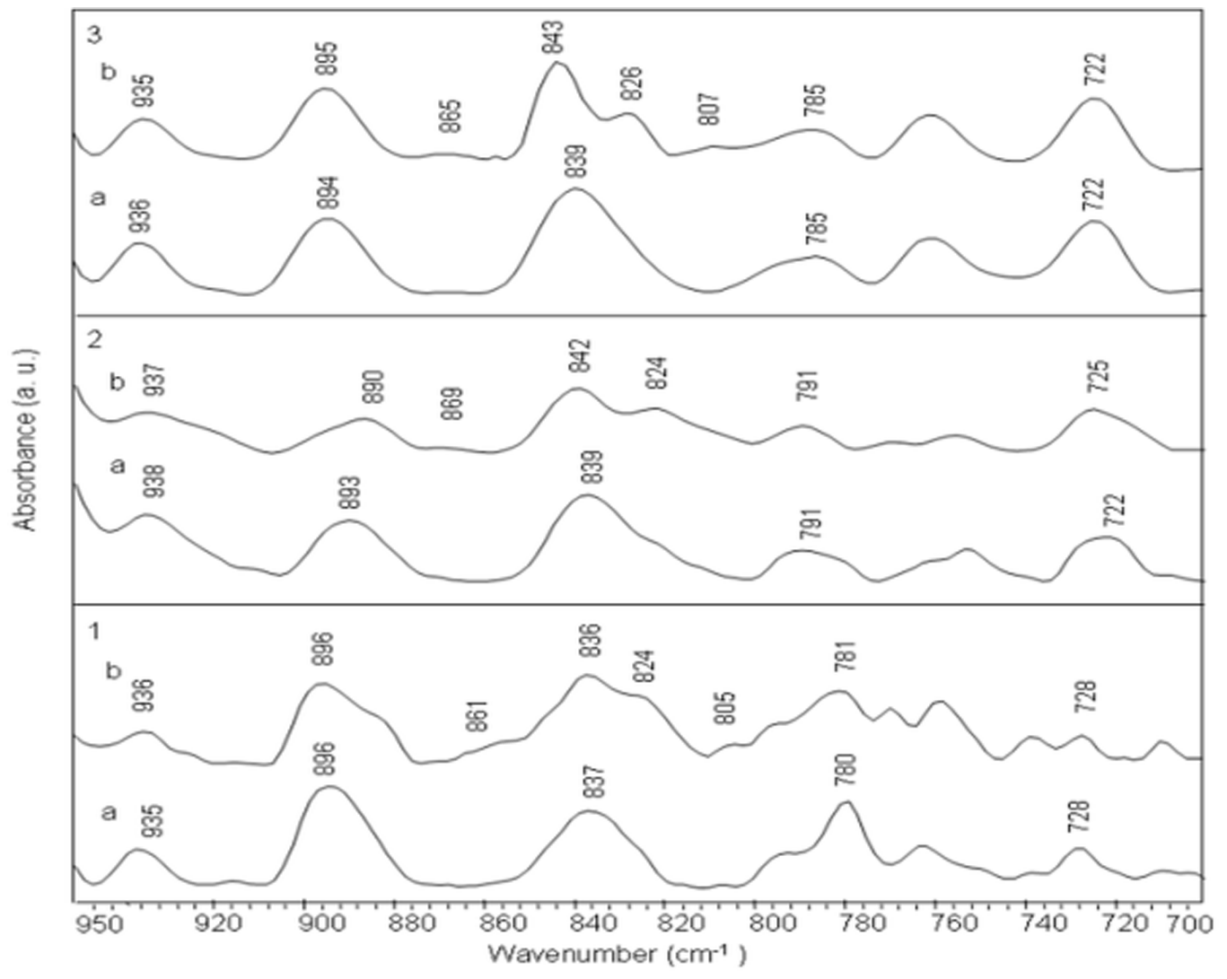
Close-up view of the FTIR spectra of ctDNA and duplexes with their complexes of amsacrine at 1/10 molar ratio (drug/nucleotide) in the 950-700 cm^−1^ spectral range. Panel 1: ctDNA (a) and complex (b), Panel 2: (AAAAAAAAAAAA)2 duplex (a) and complex (b), Panel 3: (ATATATATATAT)2 duplex (a) and complex (b), Panel 4: (TTAATTAATTAA)2 duplex (a) and complex (b), Panel 5: (AGAGCAACAGAG)2 duplex (a) and complex (b), Panel 6: (AGAGACCAGAGA)2 duplex (a) and complex (b).

Phosphate asymmetric and symmetric stretching bands (1224 cm^−1^ and 1086 cm^−1^ respectively) and sugar stretching band at 1052 cm^−1^ do not show appreciable shift in complexes, though at high drug ratios, some variations are observed. Infrared band at 969 cm^−1^ and 935 cm^−1^ are assigned to DNA backbone and considered as B-form markers of DNA (Figure 2, Figure 4 panel 1). We observe slight deviations for these two bands in the amsacrine-ctDNA complexes. Deoxyribose sugar ring vibrations produce a band at 896 cm^−1^ which shifts to 895 cm^−1^ in amsacrine-ctDNA complexes. Band at 837 cm^−1^ is the sensitive marker band of sugar pucker in S (C2′-endo/anti) type conformation (global B-DNA conformation) [31]. This band splits into two bands at 836 cm^−1^ and 824 cm^−1^ in the complexes (Figure 3, 4 panel 1). Formation of the new band at 824 cm^−1^ is indicative of some N type (C3′-endo/anti) sugar conformation (A-DNA conformation) features in amsacrine-ctDNA complexes [31], [33]. Occurrence of N type sugar conformation is further confirmed by the presence of a shoulder around 861 cm^−1^ in the complexes (Figure 3, 4 panel 1) [31], [34].

### Amsacrine- Duplex Complexes

#### Amsacrine-(AAAAAAAAAAAA)2 complex

**Figure 5:**
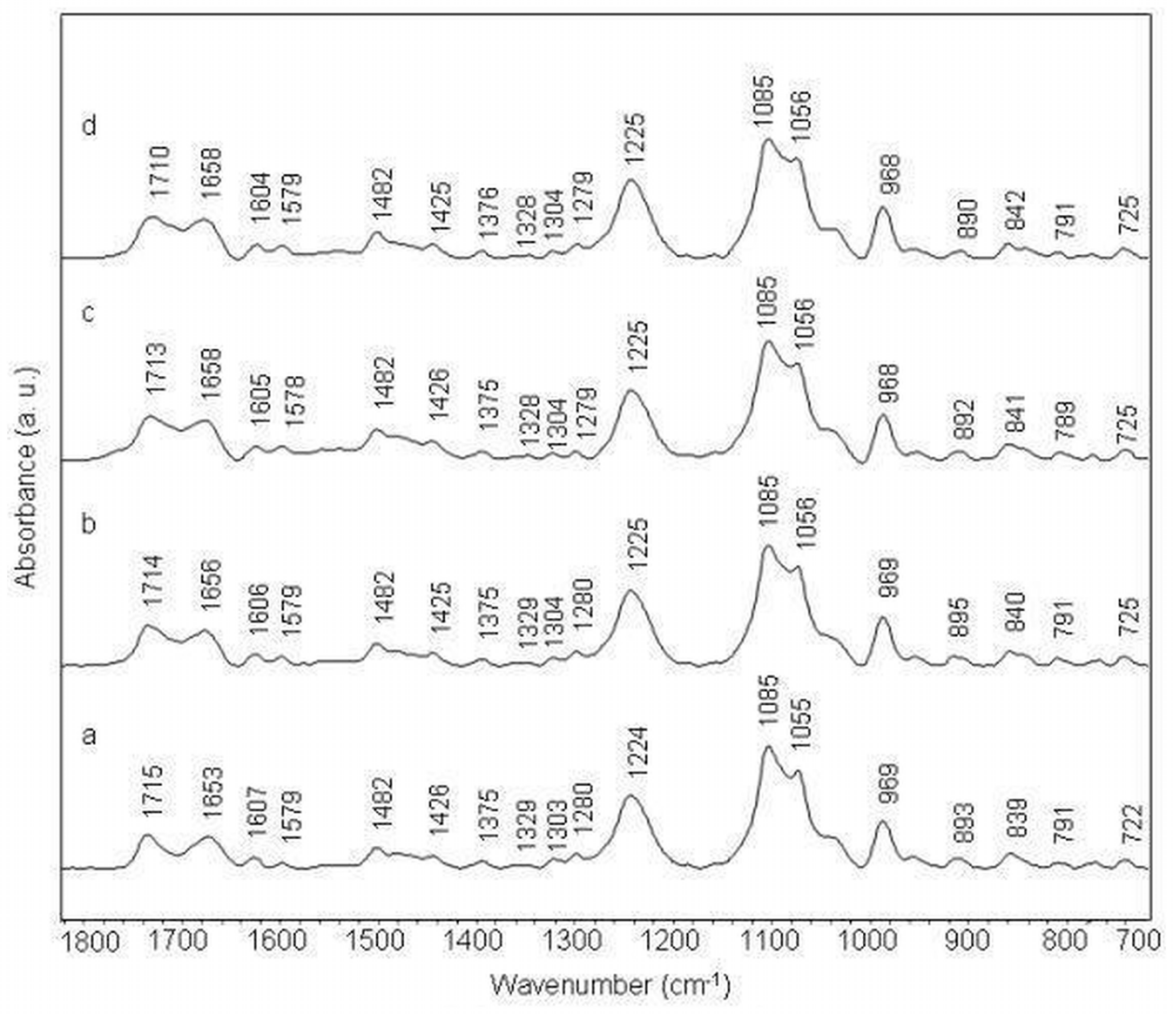
Stacked view of FTIR spectra of (AAAAAAAAAAAA)2 duplex (a) and its complexes with amsacrine at different molar ratios (r) of 1/50 (b), 1/20 (c) and 1/10 (d).

Shifts and intensity changes are observed for major bands assigned to adenine and thymine in plane ring vibrations in base stacking region. Band at 1715 cm^−1^ and 1607 cm^−1^, assigned to adenine ring vibrations, shift to 1710 cm^−1^ and 1604 cm^−1^ respectively in the complexes (Figure 5). Thymine band assigned at 1653 cm^−1^ shifts to 1658 cm^−1^ in the amsacrine-duplex complexes. Presence of new band around 1691 cm^−1^ (thymine C2 = O2 in minor groove) is apparent in second derivative spectra of complexes (Figure 3 panel 2). Minor variations are observed in second derivative spectra for the bands in base-sugar region.

IR band at 968 cm^−1^ (sugar-phosphate backbone) shows slight deviation in amsacrine-duplex complexes. IR band at 839 cm^−1^ (S type conformation) splits into two bands at 842 cm^−1^ and 824 cm^−1^ in amsacrine-duplex complexes (Figure 3, 4 panel 2). Spectral features of N type sugar pucker are more intense for these amsacrine-duplex complexes compared to amsacrine-ctDNA complexes.

#### Amsacrine-(ATATATATATAT)2 complex

**Figure 6:**
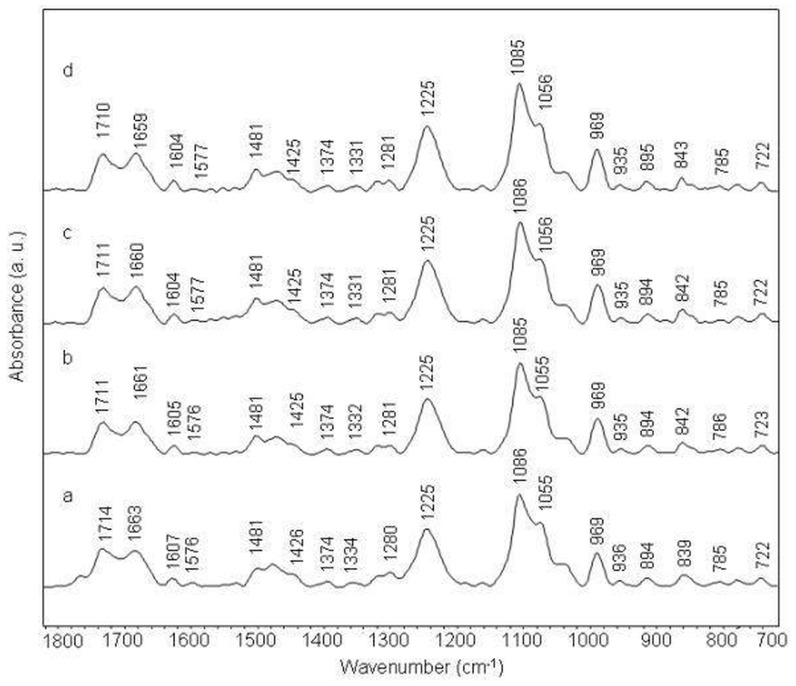
Stacked view of FTIR spectra of (ATATATATATAT)2 duplex (a) and its complexes with amsacrine at different molar ratios (r) of 1/50 (b), 1/20 (c) and 1/10 (d).

Changes observed for amsacrine-(ATATATATATAT)2 complexes are similar to those observed for amsacrine-(AAAAAAAAAAAA)2 complexes (Figure 6, Figure 3, 4 panel 3). Only difference in both the complexes comes from the base-phosphate region where added intensity variations are evident for (ATATATATATAT)2 duplex than the (AAAAAAAAAAAA)2 duplex upon interaction with amsacrine.

#### Amsacrine-(TTAATTAATTAA)2 complex

**Figure 7:**
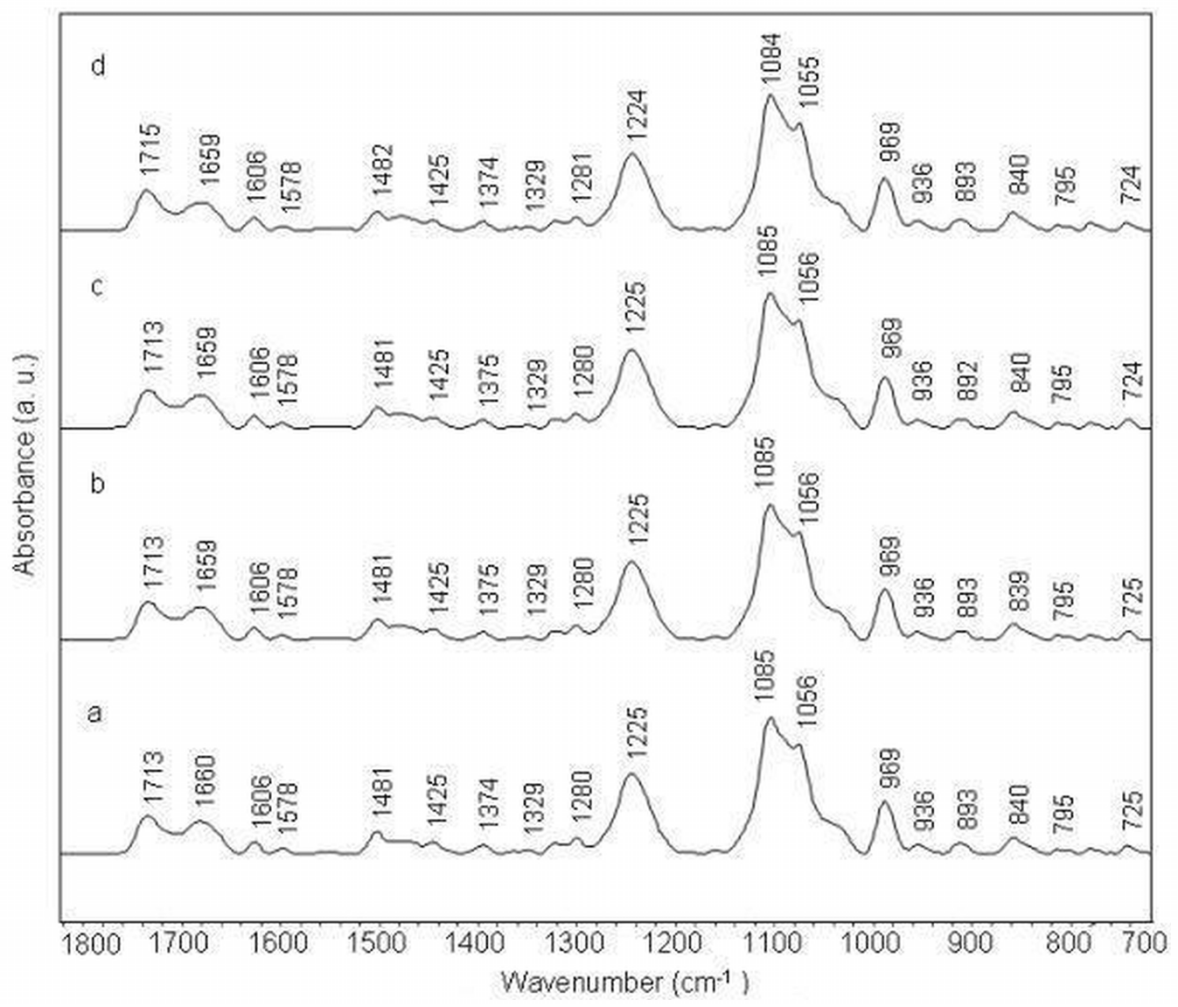
Stacked view of FTIR spectra of (TTAATTAATTAA)2 duplex (a) and its complexes with amsacrine at different molar ratios (r) of 1/50 (b), 1/20 (c) and 1/10 (d).

Compared to other complexes of amsacrine, amsacrine-(TTAATTAATTAA)2 complexes do not show any noticeable shift in band positions and intensity variation in all the major infrared bands (Figure 7, Figure 3 panel 4).

Most of the bands remain unaffected in amsacrine-(TTAATTAATTAA)2 complexes. Bands at 1713 cm^−1^, 1660 cm^−1^ and 1606 cm^−1^ show insignificant deviations even at the highest concentration used in the experiment. Consistent to other bands, sugar band at 840 cm^−1^ does not exhibit any change in the spectra of complexes (Figure 3, 4 panel 4).

#### Amsacrine-(AGAGCAACAGAG)2 complex

**Figure 8:**
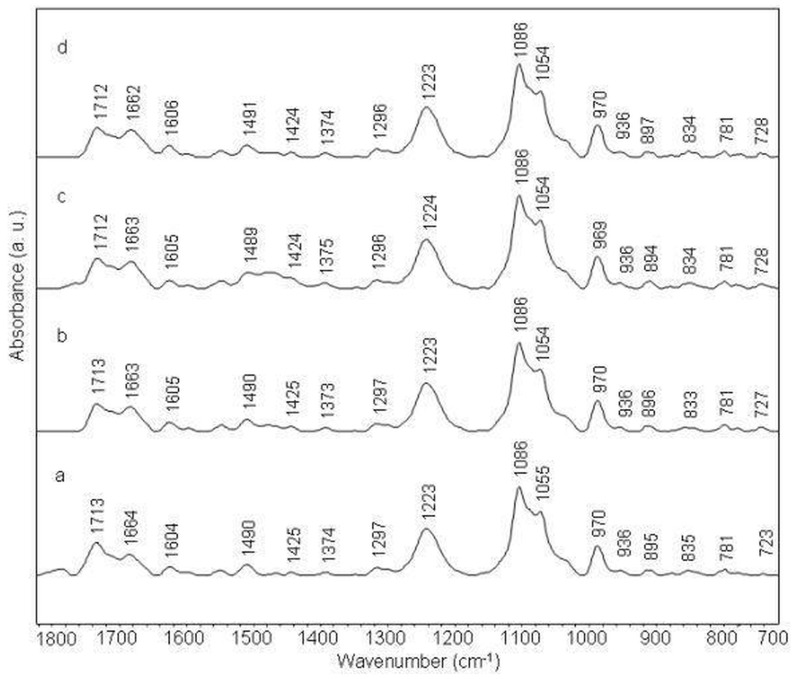
Stacked view of FTIR spectra of (AGAGCAACAGAG)2 duplex (a) and its complexes with amsacrine at different molar ratios (r) of 1/50 (b), 1/20 (c) and 1/10 (d).

IR band at 1713 cm^−1^ (guanine and adenine) marginally shifts in its position with intensity variations in the complexes (Figure 8). Adenine band at 1664 cm^−1^ shifts to 1662 cm^−1^ and thymine band at 1604 cm^−1^ shifts to 1606 cm^−1^ with some intensity changes in the spectra of amsacrine-duplex complexes. Induction of N type sugar conformation is evident by increase in the intensity of bands at 861 cm^1^ and 821 cm^−1^ in the complexes (Figure 3, 4 panel 5). Other infrared bands show negligible shift or remains unchanged.

#### Amsacrine-(AGAGACCAGAGA)2 complex

**Figure 9:**
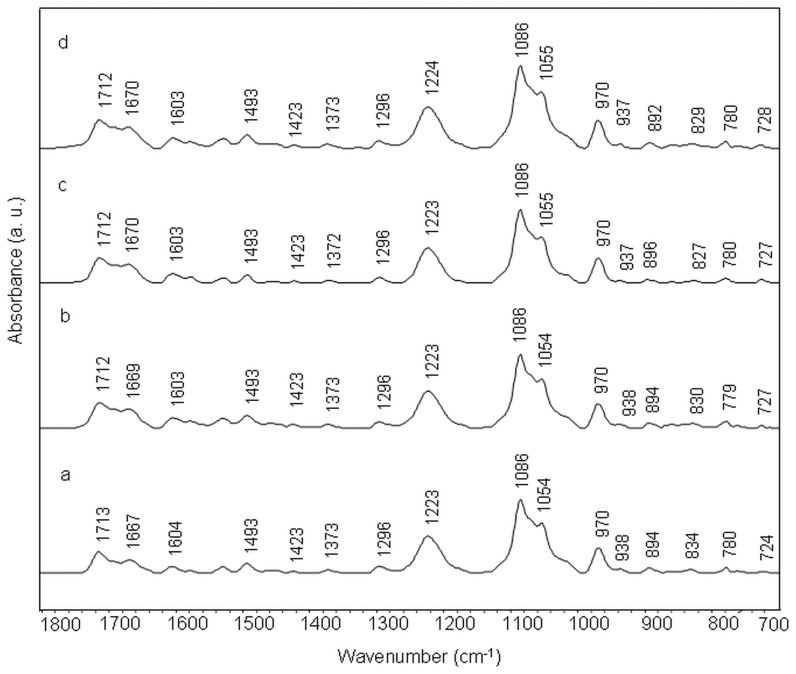
Stacked view of FTIR spectra of (AGAGACCAGAGA)2 duplex (a) and its complexes with amsacrine at different molar ratios (r) of 1/50 (b), 1/20 (c) and 1/10 (d).

This duplex shows similar variations in its complexes which was observed for amsacrine-(AGAGCAACAGAG)2 complexes (Figure 9). Some bands for example, in the sugar-base region (1500-1250 cm^−1^), show slight deviations compared to (AGAGCAACAGAG)2 duplex in the complexes (Figure 3, 4 panel 6).

## Discussion

The present study was designed to systematically evaluate the sequence and groove preferences of amsacrine with DNA. Binding preference of amsacrine for (ATATATATATAT)2 and (AAAAAAAAAAAA)2 duplexes is apparent from these ATR-FTIR spectroscopic results. Evidence for this comes from the major shift and intensity variation for the bands assigned to in-plane ring vibrations of adenine and thymine at 1715 cm^−1^, 1653/1663 cm^−1^ and 1607 cm^−1^ (Figure 5, 6, Figure 3 panel 2, 3). These changes in IR bands specify intercalation of aminoacridine chromophore of amsacrine between A/T base pairs. Intercalation is further confirmed for these duplexes by intensity changes and minor shifts in less sensitive markers for adenine and thymine at 1578 cm^−1^ and 1484 cm^−1^ (Figure 3 panel 2 and panel 3) in these duplexes. Intercalation generally affects the ring stretching vibrations of nitrogenous bases, which in turn result in intensity variations and shifting of peak positions. Changes in the thymine C2 = O2 band vibrations at 1653/1663 cm^−1^ and presence of a new band around 1692 cm^−1^ (Figure 3), further indicate the binding of amsacrine with this group in the minor groove. This band (∼ 1692^−1^) is a specific marker for minor groove interaction [35], [36]. Sulfonamide side chain of amsacrine is undoubtedly assigned as topoisomerase 2 binding domain [37], [38] and while acridine chromophore inserts between base pairs only methoxyaniline moiety remains to bind with this thymine group (C2 = O2) lying in the DNA minor groove. In the minor groove, weak interactions like hydrogen bonding and van der Waals interactions could stabilize the methoxyaniline moiety. Interestingly, in comparison to these two duplexes, amsacrine shows very weak binding with (TTAATTAATTAA)2 duplex which has the same number of A/T base pairs and differs only by their sequential arrangements. Intensity variation for band at 1660 cm^−1^ (thymine C2 = O2) is almost negligible for amsacrine-(TTAATTAATTAA)2 complexes compared to other two duplexes (Figure 7, Figure 3 panel 4). Consistent to other bands, sugar pucker marker bands also remains unaffected in its complexes with amsacrine (Figure 3, 4 panel 4). this differences in interaction arises due to differences in groove structures and geometries. Sequences that contain four or more AAAA or ATAT are classified as A-tracts or A-steps. These A-tract sequences comprise narrow minor groove with single or double spine of bound water [39]. A-tracts have negative roll angles and propeller twists, which further compress the minor groove [39]. these sequences are highly favored by minor groove binders. In contrast to A-tracts, steric clash of the cross-strand adenines occurs in TA steps (in TTAA and TATA repeat sequences), which widens the minor groove [39], [40]. Therefore, TA steps are disruptive for minor groove interaction by ligands. It is established that different minor groove binding ligands interact with AAAA and AATT sequences much stronger than TTAA and TATA sequences [41]. Due to weak van der Waals interactions, binding enthalpy should be inferior for shallow minor grooves of TTAA repeat sequence compared to deep grooves of ATAT and AAAA sequences. Moreover, lack of spine of bound water is also an important factor, which precludes binding of minor groove ligands with TTAA repeat sequences. Minor groove binders are highly specific about groove structure and their hydration pattern, therefore differential binding of amsacrine with (AAAAAAAAAAAA)2/(ATATATATATAT)2 and (TTAATTAATTAA)2 suggests the position of methoxyaniline moiety in the minor groove. Methoxyaniline moiety provides discriminating factor of amsacrine between different minor grooves while acridine chromophore confers A/T base pair requirement for intercalation and acts as DNA anchor of amsacrine. Both constituents (methoxyaniline and acridine chromophore) augment the binding specificity of amsacrine.

These findings are consistent with the observation that DNA binding affinity of aminoacridine compounds are unaffected by blockage of the major groove of the DNA while marked decrease in their binding affinity was observed when minor groove binding agent is added [42]. Amsacrine binding does not perturb phosphate/phosphodiester vibrations of ctDNA and duplexes. It is apparent by observation of phosphate and phosphodiester bands in the 1250-1000cm^1^ region, which do not exhibit any significant deviations. It implies that amsacrine orients itself in such a manner that methoxyaniline moiety is away from backbone and simultaneously interacts with walls of minor grooves and thymine C2 = O2 group. In such a geometric orientation, where methoxyaniline group is perpendicular to the plane of acridine chromophore [43] and both moieties interact with base pairs and minor groove; deep penetrations of base pairs is not possible [20]. This explains why amsacrine is a weak DNA binder. A QSAR (quantitative structure-activity relationship) study done by Denny et al. further supports that groove-binding mode is essential for biological activity of amsacrine [44]. Earlier studies show that c*-myc* protooncogene is 20 times more susceptible for amsacrine-mediated inhibition than global genome [16]. Topoisomerase 2 binding sites are preferentially found in c*-myc* transcription regulatory regions. Therefore, differences in DNA sequence selectivity exhibited by amsacrine here may contribute to the selective antitumor activity of topoisomerase 2 inhibition (e.g. inhibition of c*-myc* gene). Amsacrine binds with ctDNA in similar fashion as with (AAAAAAAAAAAA)2 and (ATATATATATAT)2, though DNA binding is weaker compared to these duplexes. It is obvious by the given fact that (AAAAAAAAAAAA)2 and (ATATATATATAT)2 offer better minor groove which could accommodate methoxyaniline moiety in a better way than groove of random sequence of ctDNA. Amsacrine binding with (AGAGCAACAGAG)2 and (AGAGACCAGAGA)2 sequences are weak due to the presence of alternating guanine and adenine, which creates wider minor groove. Moreover, guanine amino groups approaching in minor groove of these sequences also hinder amsacrine interaction [45]. Binding preference of amsacrine for all sequences studied here is found as: (AAAAAAAAAAAA)2 ≈ (ATATATATATAT)2> (ctDNA)>(AGAGCAACAGAG)2 ≈ (AGAGACCAGAGA)2> (TTAATTAATTAA)2.

### Effects of Amsacrine Binding on Local Conformation of DNA

Antisymmetric PO_2_ stretching band (1225 cm^−1^) is a marker for DNA in B conformation, regardless of base vibrations and sugar pucker [31], [32]. This band did not show any appreciable shifts in the spectra of complexes. As aforementioned, IR band at 839 cm^−1^ is a fairly sensitive measure of S (C2′-endo/anti) sugar pucker and thus indicative of local DNA conformation. S (C2′-endo/anti) type sugar pucker corresponds to global B-DNA conformation. Split into this band (∼842 and ∼824 cm^−1^) and presence of new shoulders around ∼865and ∼808 cm^−1^ in the complexes (Figure 3, 4) are indicative of some N type (C3′-endo/anti) sugar features in complexes [31], [33], [34]. N (C3′-endo/anti) type sugar pucker represents A-DNA conformation. Hence, these features signify changes in local conformation of DNA and coexistence of two different major sugar puckers (conformations) upon interaction with amsacrine. Amsacrine induces similar kind of structural changes in different sequences regardless of their composition. It implies that rigid structure of amsacrine induces local conformational changes in DNA to accommodate itself between base pairs. In a recent study, similar results on conformational alternations in DNA structure were observed for 9-amino-DACA, which alike to amsacrine, is an aminoacridine derivative [46]. Conformation alternations induced by amsacrine may change the major/minor groove ratio in the vicinity of amsacrine intercalation and hence could bend or kink the DNA at these sites. These fluctuations in DNA structure could be of high importance because they may be sensed by topoisomerase 2 and hence impart in formation and stabilization of ternary complexes. It is proven by recent *in vivo* studies that DNA kinks and bend have important role in the DNA binding by topoisomerases and thus they create preferred binding sites for the enzyme [47].

During Intercalation, planer chromophore slides between two base pairs without breaking Watson-Crick hydrogen bonds. Chromophore of an intercalator interacts with adjacent base pairs through pi stacking. These interactions have a stabilizing effect on DNA’s structure, which leads to a raise in its melting temperature. In groove binding mode, hydrogen bonding, Van der walls and ionic interactions etc. occur between ligand and functional group(s) of DNA. This mode of interaction also exerts stabilizing effects on DNA structure. So overall, amsacrine interaction results in enhanced stability of DNA helical structure. Although amsacrine interaction results in local conformation change in DNA, globally DNA helix remains in native B-DNA conformation. This is clear by no appreciable changes (shift and intensity) in global conformational marker bands at 1225 cm^−1^ and 970 cm^−1^ in the spectra of complexes.

### Conclusions

Our results suggest that mixed-ligand type binding takes place between amsacrine and DNA. These ATR-FTIR spectroscopic analysis demonstrate that amsacrine binding with DNA requires an A/T base pair and suitable minor groove and it is less sensitive to direct read-out of DNA, therefore all AT rich sequences are not equally favored. Minor groove interaction of amsacrine should play a vital role in its cytotoxicity. Results obtained here for amsacrine could be extended to other related anilinoacridines which share amsacrine like pharmacophore.
