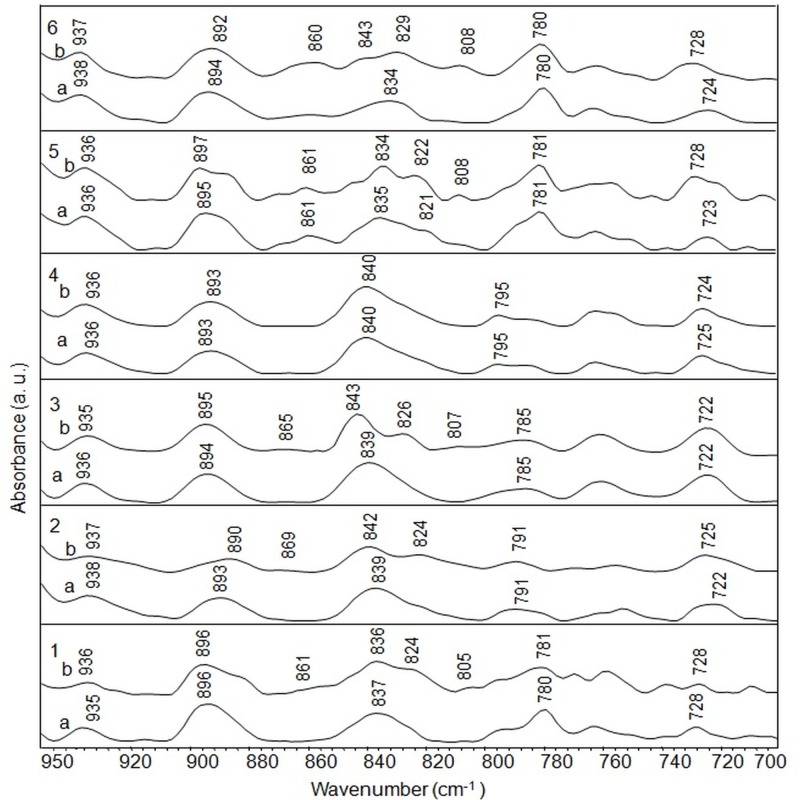# Correction: Role of Minor Groove Width and Hydration Pattern on Amsacrine Interaction with DNA

**DOI:** 10.1371/annotation/bd6354ef-300a-42cc-9e38-9dad6685a04c

**Published:** 2013-12-13

**Authors:** Deepak K. Jangir, Suman Kundu, Ranjana Mehrotra

The incorrect Figures 3 and 4 were erroneously included in the article. Please see the correct Figures 3 and 4 here:

Figure 3: 

**Figure:**
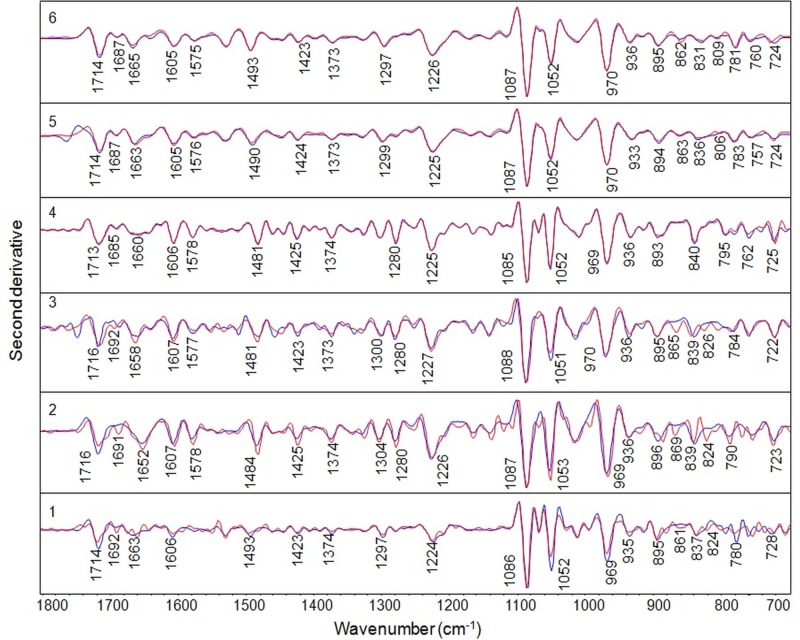


Figure 4: 

**Figure:**